# Research Progress Related to Aflatoxin Contamination and Prevention and Control of Soils

**DOI:** 10.3390/toxins15080475

**Published:** 2023-07-25

**Authors:** Xue Wang, Dun Wang, Shujuan Zhang, Mengjie Zhu, Qing Yang, Jing Dong, Qi Zhang, Peng Feng

**Affiliations:** 1 Xiangyang Academy of Agricultural Sciences, Xiangyang 441057, China; maysxuer@126.com (X.W.); zmj2280880980@163.com (M.Z.); qingyunsanhao@126.com (Q.Y.); xynkydongjing@163.com (J.D.); fengpengxynky@163.com (P.F.); 2 Oil Crops Research Institute, Chinese Academy of Agricultural Sciences, Wuhan 430062, China; zhangqi01@caas.cn; 3 Hubei Hongshan Laboratory, Wuhan 430061, China; 4 Zhejiang Mariculture Research Institution, Wenzhou 325000, China; zhangshujuan1990@126.com

**Keywords:** *Aspergillus flavus*, aflatoxin, soil, aflatoxin contamination, aflatoxin control, ARC-BBBE

## Abstract

Aflatoxins are potent carcinogenic compounds, mainly produced by fungi species of the genus *Aspergillus* in the soil. Because of their stability, they are difficult to remove completely, even under extreme conditions. Aflatoxin contamination is one of the main causes of safety in peanuts, maize, wheat and other agricultural products. Aflatoxin contamination originates from the soil. Through the investigation of soil properties and soil microbial distribution, the sources of aflatoxin are identified, aflatoxin contamination is classified and analysed, and post-harvest crop detoxification and corresponding contamination prevention measures are identified. This includes the team’s recent development of the biofungicide ARC-BBBE (Aflatoxin Rhizobia Couple-*B. amyloliquefaciens*, *B*. *laterosporu*, *B*. *mucilaginosus*, *E*. *ludwiggi*) for field application and nanomaterials for post-production detoxification of cereals and oilseed crops, providing an effective and feasible approach for the prevention and control of aflatoxin contamination. Finally, it is hoped that effective preventive and control measures can be applied to a large number of cereal and oilseed crops.

## 1. Introduction

Aflatoxin is produced by *Aspergillus flavus*, which is classified as a Group 1 carcinogen by the International Agency for Research on Cancer (IARC). It is one of the most toxic compounds known, acting mainly on human and animal liver tissues, capable of inducing cancer of the liver (primarily), as well as the pancreas, kidney, bladder and other organs. Aflatoxin may also lead to malnutrition, immunosuppression, and other pathologies with mutagenic, hepatotoxic, and nephrotoxic outcomes [1,2]. Aflatoxin mainly contaminates grain and oil crops, feed, nuts, Chinese herbs and other crops, and then contaminates meat, eggs, milk and other by-products after being ingested by animals. The import and export of agricultural and sideline products all over the world have strict limits on aflatoxin, thus restricting industrial development and export trade. Aflatoxin contamination not only causes huge economic losses to food crops, but also has a negative impact on the health of consumers. According to the data from the Food and Agriculture Organization of the United Nations (FAO), about 25% of crops worldwide are contaminated with moulds and their toxins each year, while about 2% of agricultural products lose their value due to excessive toxin contamination [3], such as the 100,000 turkey deaths that first occurred in the 1960s in the UK, and the high annual economic losses caused by aflatoxin contamination in peanuts in Georgia, USA [4].

There have been many cases of human and animal mass poisonings caused by aflatoxin contamination of agricultural products and foodstuffs all over the world. Aflatoxin is highly toxic to the liver and central nervous system of humans and animals. It can cause acute poisoning or even death in humans and animals when ingested in large amounts at one time and can be teratogenic, mutagenic, and carcinogenic when ingested in small doses over a long period of time [5,6]. According to the IARC, about 500 million people in the developing world alone are still at risk of aflatoxin exposure [7]. The European Union, one of the economies with the best food safety management systems today [8], has strict limits for fungi toxin contamination in food and feed, and China also has strict limits for aflatoxin B1 in food (Table 1). The Chinese GB 2761-2017 “National Standard for Food Safety Limits for Mycotoxins in Food” requires that the maximum limits for aflatoxin B1 (AFB1) in different cereal products range between 5–20 µg/kg, while the maximum limit for AFB1 and aflatoxin M1 (AFM1) in special dietary foods is 0.5 µg/kg and should not be detected in infant diets [9].

Therefore, research on the prevention, control and detoxification of aflatoxins in food and feed has become one of the most important aspects of food safety and has attracted widespread attention. In order to prevent and control aflatoxin contamination from the source of crop production, improve the quality and safety of agricultural products in China, and ensure consumer safety and healthy development of the agricultural industry, the source, nature and contamination pathways of aflatoxin, and the current effective methods to deal with aflatoxin in crop production were summarized in this paper. It is expected that the emerging new technologies for aflatoxins control in soils will be widely used in crop production.

## 2. *Aspergillus flavus* in Agricultural Soils

There are an estimated 7000 species of fungi that inhabit the soil [10]. Luo et al. [11] studied the rhizosphere soil fungi community composition of camellia and explored the correlation between rhizosphere soil fungi and soil environmental factors, concluding that camellia diseases could be prevented by regulating soil environmental factors. Wu et al. [12] studied the fungi community structure in the rhizosphere soil of Rehmannia varieties and found that changes in the number of some common fungi pathogens such as *A. flavus* and *Aspergillus niger* might be the cause of soilborne diseases in the soil, which suggests that the Rehmannia root system had a certain plastic ability to the number, composition and species of fungi in rhizosphere soil.

So far, there are few reports on soil fungi of grain and oil crops. Due to the limitation of separation and detection technology in soil, only species suitable for artificial environments can be isolated from soil, so it cannot fully reflect the real soil colony environment. Fungi isolated from soil can be cultured. Only propagules capable of growing and sporing on the isolated medium used can be detected, and only about 17% of known fungi species can be successfully grown in the culture at present [13].

A single fungus may produce multiple mycotoxins, and a toxin may also be produced by multiple fungi; there are over 150 species of fungi that can produce one or more of 300 potential mycotoxins. As fungal growth is geographically specific, the predominant mycotoxins vary from region to region, e.g., in subtropical and tropical regions, agricultural products and feed are mainly contaminated with aflatoxins and certain ochratoxins. *A. flavus* was first used by LINK in 1809 as a generic term for *saprophytic moulds* in soils [14]. It has a wide range of hosts and has been reported in agriculture on maize, rice, wheat, cottonseed, peanuts and nuts, with peanuts and maize being the most affected [15,16]. The aflatoxin-producing fungi in the soil are diverse, and the distribution characteristics of different toxin-producing *A. flavus* occur differently. So far, the infestation pathways, effects and field distribution characteristics of *A. flavus* as the source of aflatoxin production in soil have not been systematically studied.

*Aspergillus flavus* is widely present in soil. According to the data from FAO, *A. flavus* is one of the most important contaminating fungi of cereals worldwide [4]. The optimum growth temperature for *A. flavus* ranges from 12 °C to 34 °C, while the optimum toxicity-producing temperature ranges from 20 °C to 30 °C, within 45 °C latitude [17,18]. *A. flavus* is mostly distributed in the soils of the Yangtze River basin and has the greatest risk of contamination [19]. Studies on the distribution of soil microbial flora and toxin contamination have also been carried out all over the world. Soil type is also related to aflatoxin pollution to some extent. According to different soil, the distribution of microbial flora is different, and the degree of mycotoxin pollution is also different, so targeted prevention and control measures can be taken. Wei et al. [20] found the existence of non-toxic and toxin-producing *A. flavus* in peanut soil. Based on the distribution characteristics of the strains, the risk of toxin contamination in different producing areas of China was evaluated. Zhang et al. [21] studied the genetic characteristics of *A. flavus* in peanut soil, providing a technical basis for the later screening of non-toxic strains and the development of aflatoxin biocontrol fungi.

In recent years, only the isolation and screening of aflatoxin in peanut root soil has been reported. Yang et al. [22] predicted the aflatoxin contamination of postpartum peanuts based on the number of aflatoxin colonies in the soil of four peanut-producing areas in China, so as to ensure the prevention and control of aflatoxin in peanuts in the later period. Zhang et al. [19], Zhu et al. [23] studied the distribution, toxin production and aflatoxin infection of *A. flavus* in the soil in the main peanut-producing areas of China, which provided a theoretical basis for the establishment of a model for the prevention and control of aflatoxin in China. According to the analysis of aflatoxin and its virulence in 11 producing areas of China, the Yangtze River Basin has the largest distribution of aflatoxin and the greatest risk of aflatoxin pollution. Because of the unique climatic conditions and geographical environment of the Yangtze River Basin, Hubei province has also become the largest peanut production area in China. Zhu et al. [24] also studied the distribution and toxic characteristics of aflatoxin in the soil of typical peanut growing areas in Hubei Province, providing a theoretical basis for the establishment of the early warning and prevention model of aflatoxin pollution of peanuts in Hubei Province. Zhang et al. [25] first discussed the relationship between soil types and *A. flavus* colonies in the peanut production area of Xiangyang, Hubei province. This work suggested that the number of *A. flavus* groups in the clay loam was higher and the virulence was higher than that in the sandy loam, while the sandy loam had a smaller distribution density and infection risk of *A. flavus* under appropriate irrigation conditions. The results of this study have important guiding significance for field fertilization, irrigation, *A. flavus* control and other agronomic management in the local peanut planting process.

## 3. Aflatoxin Contamination

So far, the content of mycotoxin detected in the soil is all in the μg range. For example, the maximum content for zearalenone is 72.1 μg/kg, for deoxynivalenol is 32.1 μg/kg, for ochratoxin A is 23.7 μg/kg, for nivalenol is 6.7 μg/kg, and for aflatoxin is 5.5 μg/kg. The retention of mycotoxins in soil is affected by soil type. Clay soil is easy to absorb toxin compounds, but sandy soil has the potential to leachate compounds [26]. In 1980, C^14^ was used to label aflatoxins to analyze the decomposition rate of aflatoxins in soil. Since microbial degradation function existed in soil, AFB1 could not be detected after 77 days [27]. Hence, the pollution risk of aflatoxin in soil was low, and its main risk was in the storage period after harvest. In 1997, the first report on *Aspergillus oryzae* detected in water storage tanks showed that although it was not drinking water, there was still a risk of potential mycotoxin contamination [28]. Although mycotoxins in freshwater samples have been increasingly reported, no reports of mycotoxins in detected sediments were found. Accinelli et al. [29] studied aflatoxin residues in soil and corn crops and proposed that AFB1 could degrade quickly in a 28.8 °C soil environment (half-life is 5 days), and AFB1 was mainly produced by the residues of corn crops on the soil surface. Corn residues may be an important source of aflatoxin pollution in soil. Therefore, if maize returning to the field in late harvest is effectively controlled, the aflatoxin pollution will be greatly reduced. In general, soil and sediment are still under-represented in the study of mycotoxin potential for environmental contamination.

Aflatoxins are mainly produced by toxic fungi such as *A. flavus*, as well as *Aspergillus parasiticus* and *Aspergillus nomius*. Aflatoxin-contaminated soil and agricultural products, especially grain and oil crops and nuts, are at the greatest risk of contamination. Khan et al. [30] believed that soil was the main source of aflatoxin contamination of crops. Tran-dinh et al. [31] studied *A. flavus* in Vietnamese soil and found that all the isolated *A. flavus* came from cultivated soil. Aflatoxin pollution is mainly concentrated in *A. flavus* (Table 2), which comes from the soil. Soil fungi are an important part of soil microorganisms. Horn et al. [32] proposed that climate and crop composition affect colony density and aflatoxin toxicity. *A. flavus* exists in soil in the form of conidia, sclerotia and mycelia, as the main inoculum for direct infection of peanuts or above-ground crops. Peanut is a crop with a lot of aflatoxin infection since the peanut shells are in direct contact with the soil. Aflatoxin pollution in peanuts mainly comes from the soil *Aspergillus*. In the study of the rhizosphere soil, through the dynamic analysis of the soil *Aspergillus*, it is of great significance to discuss the prenatal prevention and control of aflatoxin pollution.

Many studies have shown that soil is the main source of aflatoxin pollution in most rhizosphere crops [33,34,35], and the direct contact between soil and plant roots and the exchange of nutrients have a great impact on the occurrence of aflatoxin pollution in crops [36]. There are many research studies on soil microbial flora, but the investigation of *A. flavus* in soil is less. It was also reported that different soil types and *A. flavus* had different distributions, and the virulence of the strain was also very different, thus affecting the aflatoxin pollution of crops [37,38]. Coupling studies can change the population structure through crop rotation and management methods [39]. Horn and Dorner [40] studied the *A. flavus* strains in the soil of peanut planting in some areas where cotton is widely grown in the United States. In some studies, it has been possible to control aflatoxin contamination by adjusting crop rotations and changing soil temperatures.

**Table 2:** Sources and contamination of toxins in food crops.

Toxin	Fungus	Susceptible Crop(s)
aflatoxins	*Aspergillus* spp.	peanut, soybean, maize, etc. [41]
zearalenones	*Fusarium* spp.	wheat, oats, etc. [42]
ochratoxins	*Aspergillus* spp. *Penicillium* spp.	wheat, maize, rice, soybean, etc. [43]
trichothecenes	*Fusarium* spp.	wheat, oats, maize, etc. [44]
fumonisins	*Fusarium* spp.	maize [45]

**Figure 1:**
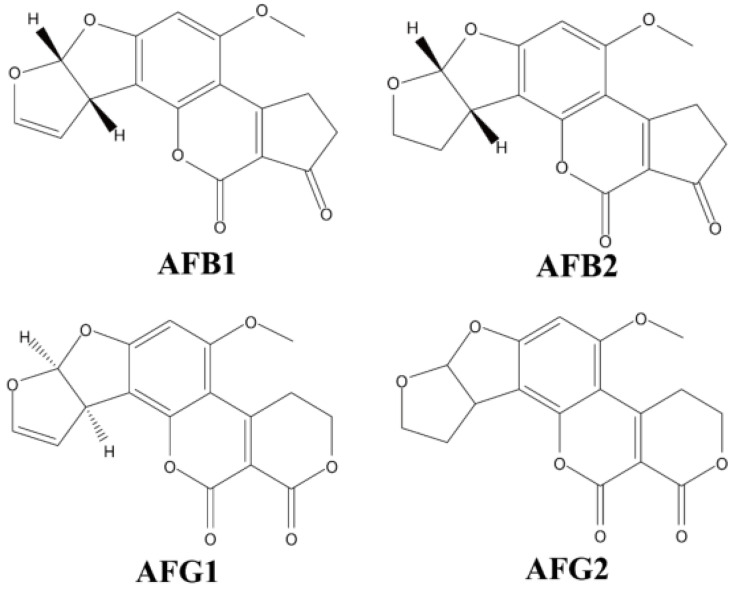

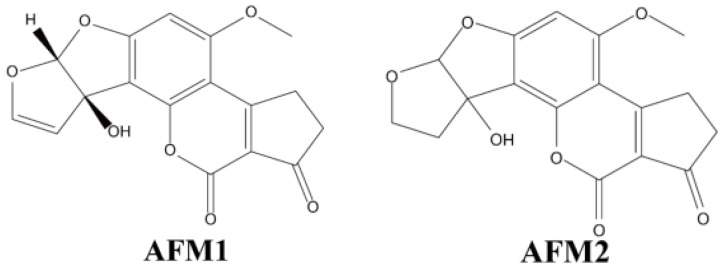
Chemical structures for six main types of aflatoxin.

Aflatoxin is a secondary metabolite produced by multiple *Aspergillus* species. It is colourless, odourless, and extremely toxic. It has been well studied that its chemical structure includes coumarin and difuran rings, and it has many derivatives and isomers that have been well studied [46]. B aflatoxins are so named because they fluoresce blue, while G aflatoxins fluoresce green when exposed to long-wave UV light (365 nm) [4]. Only 50% of *A. flavus* strains produce aflatoxins and B aflatoxins only, whereas almost all *A. parasiticus* strains produce both group B and G aflatoxins (Figure 1) [47]. The main forms of aflatoxins present in crops are AFB1, AFG1, AFB2 and AFG2, with toxicity being AFB1 > AFG1 > AFB2 > AFG2. Among them, AFB1 has the most stable structure and AFB1 is classified as a class I carcinogen [22]. International European standards have limits of AFB1 ≤ 2 µg/kg and total aflatoxin (AFT) must not exceed 4 µg/kg [48]. According to Chinese standard GB2761-2017 [9] “Food Safety National Standard Food Mycotoxin Limits”, the maximum contain limitation for AFB1 is 20 µg/kg in peanuts, corn and their products, 10 µg/kg in rice and oils, and 5 µg/kg in grain, beans, fermented foods and condiments, etc.

According to the relationship between virulence and the size of its sclerotia, *A. flavus* can also be divided into L and S types. The S-type strains produced numerous small (<400 μm) sclerotia, while the L-type strains produced fewer, larger sclerotia [49]. Most of the L types are non-aflatoxigenic, while most of the S-type strains are highly toxic [50,51]. Crop rotation and soil temperature can also affect the distribution of fungi community structure. Ramon et al. [52] found that the number of *A. flavus* and the proportion of S-type strains increased with soil temperature. Therefore, we can control *A. flavus* pollution by changing soil temperatures and crop rotation.

## 4. Aflatoxin Pollution Prevention and Control Measures

Aflatoxin contamination of crops predominantly comes from the soil and is not uncontrollable. Relevant prevention and control measures have also been studied and reported in the past two years. One approach involves a post-harvest perspective, whereby rapid detoxification and use of the product reduce the loss of marketable agricultural products. This would use physicochemical and biological methods to degrade or adsorb aflatoxin (Table S1), such as the adsorption method, radiation method, ultraviolet method, fumigation method and/or microbial enzyme degradation method [53]. These methods are relatively simple, quick, easily replicated and efficient; however, there is a risk of waste should the detoxification be unsuccessful. An alternative perspective, pre-harvest prevention, reduces aflatoxin pollution through (1) improvement of the soil microenvironment, thereby reducing the distribution of aflatoxin-producing strains, or (2) establishment of a mechanism of control in advance of planting aflatoxin-susceptible crops. Pre-harvest control is achieved through the use of biological agents acting on the soil, thereby changing the proportion of microbial strains in the soil. For example, adding ARC-BBBE biofungicide to the soil can reduce the distribution of aflatoxin colonies in peanut soil, thus reducing the total aflatoxin content of peanuts after production [51]. Biological control methods are better for maintaining the original raw material’s nutritional value and are mild, irreversible and economically viable. However, the living organisms used as agents can be influenced by the environment and have the potential to alter the soil environment in unwanted ways. A third perspective involves the establishment of an early warning model to be used by growers of crops affected by aflatoxin contamination ahead of planting, so that growers know if there is a potential risk of aflatoxin contamination. The modelling system is safe and effective in the long-term; however, there are limitations, such as being restricted by regions with geographical differences. Currently, there are very few aflatoxin modelling systems in use [54,55].

### 4.1. Aflatoxin Prevention and Control Using Biological Agents

Some soil biological control agents use competitive growth, or the secretion of secondary metabolites, to inhibit growth and/or toxin production by *A. flavus.* Examples of effective microorganisms include fungi such as *Aspergillus niger* and non-aflatoxigenic *A. flavus*, as well as lactic acid bacteria (*Lactobacillus* spp.) [56].

#### 4.1.1. Non-Aflatoxigenic *Aspergillus* Strains

Dorner et al. [57] started to study the feasibility of non-toxic producing fungi for the control of aflatoxin contamination in peanut cultivation in 1992 with satisfactory results. Researchers such as Horn [58], Cotty [59] and Abbas [60] investigated the effectiveness of different non-aflatoxigenic *A. flavus* strains as formulations for the biological control of aflatoxin in peanut, cotton and maize fields, respectively. In these studies, the mechanism of control reportedly used by non-aflatoxigenic *A. flavus* strains to inhibit the growth of toxin-producing *Aspergillus* strains was competitive exclusion. Mark et al. [61] have found that field inoculation with inhibitory strains can reduce the probability of *A. flavus* contamination in both pre- and post-harvest. In the field, spore preparation of 11.2–22.4 kg/hm^2^ can inhibit aflatoxin in peanut crops by up to 90%, and this inhibition effect can still be sustained. In this method, non-aflatoxigenic *A. flavus* strains are used to inhibit the growth of toxin-producing *A. flavus* strains in soil, while non-aflatoxigenic strains in crops have a certain protective effect on crops after harvest [62]. Liu et al. [63], Xing et al. [64] and Zhang et al. [65] studied several strains of non-aflatoxigenic *A. flavus* and their roles in aflatoxin degradation, and the inhibition levels in the laboratory reached 98%. No finished formulations have yet been applied to field soils, and research has mainly focused on the screening and optimisation of biocontrol fungi in the laboratory and the investigation of control mechanisms.

#### 4.1.2. Yeasts

In 2022, Natarajan et al. [66] isolated 45 strains of yeast from the soil to inhibit the growth of *A. flavus*, and the inhibition rate reached 99% in the laboratory, but it was not applied in the field. Biological control of yeasts is widely used for post-production detoxification, using their adsorption capacity to remove aflatoxins from food, and there are no live strain preparations that have been applied in actual field trials [67].

#### 4.1.3. Bacteria

In 1985, Coallier-Ascah et al. [68] inoculated *Lactococcus lactis* into a culture of aflatoxigenic *A. flavus* spores and did not detect aflatoxin after shaking bed incubation. In 2008, Petchkongkaew et al. [69] used *Bacillus subtilis* and *Lactobacillus licheniformis* to inhibit the growth and toxin production of aflatoxin and both achieved good control results. In recent years, Zhou et al. [51] studied ARC-BBBE biological bacteriological agents and applied three species of *Bacillus* to the rhizosphere soil. During three years of field demonstration in major peanut-producing areas in China, the abundance of toxic *A. flavus* in soil decreased by 66.5%, the detection rate of aflatoxin in post-harvest peanuts was also greatly reduced, and nodulation with nitrogen fixation of root system was discovered unexpectedly. Large field demonstration trials have achieved substantial yield increases.

### 4.2. New Material Detoxification Methods

With the rapid development of materials, biology, environment and energy science, it is the direction of future efforts to find a low-cost, fast, safe, efficient and stable green technology for aflatoxin detoxification in grain and oil by combining the above technologies effectively. Nanomaterial scales are equivalent to 10 to 100 atoms packed tightly together. It has been reported that nanomaterials can be used for the elimination of aflatoxins [70]. By modifying nanomaterials with surface modification, nanomaterials with specific adsorption of aflatoxins can be utilized effectively. Liang et al. [71] analysed the feasibility of the magnetic nanoparticles selective adsorption method and tested it for the detoxification of aflatoxin in peanut oil. In the later stage, Mao et al. [70,72,73] studied the semiconductor material g-C3N4, which could be degraded to carbon dioxide and water after the adsorption of AFB1 and 2 h of sunlight irradiation. They also prepared z-composites at a later stage and designed effective photocatalysts to reduce secondary contamination by aflatoxin toxicity tests on cells.

### 4.3. Early Warning Models

The crop aflatoxin production is influenced directly by environmental temperature and humidity changes at the planting, harvesting, storage, transportation and processing stages. To reduce the risk of aflatoxin pollution, early warning models are often established according to the relationships between environmental temperature, humidity changes and aflatoxin production. As early as 1990, Thai et al. [74] studied the process dynamics of aflatoxin pollution under drought conditions and established the relationship model between soil temperature and aflatoxin, but it has not been applied in practice. In 1998, the CROPGRO-peanut model was released in the United States, which comprehensively introduced the relationship between environmental parameters and the growth of *A. flavus* [75]. It was also applied to the risk warning of aflatoxin in Niger and was also well applied to the prediction of aflatoxin content in Mali [76].

Li et al. [77] established Boltzmann and logistic models to explore the relationship between temperature and humidity during storage and aflatoxin, effectively preventing contamination. Jiang et al. [78] put forward recommended measures for the whole-process prevention and control of aflatoxins based on GMP standards by pre-harvest investigations of the peanut varieties, soil, planting methods, pest control and field irrigation before flower production, as well as the harvesting equipment and post-harvest considerations like receiving time, drying and cleaning, transportation conditions and storage environment. Zhang et al. [79] analysed the causes of product hazards, critical control points and control measures from three aspects, and used HACCP to study the whole process control of aflatoxin production in exported peanuts. Wu et al. [80] studied the pre-harvest, post-harvest and whole-process early warning methods of aflatoxin, which collects data in different locations and uses different links to build models, and is also the development direction of aflatoxin early warning technology in the future.

The existing research shows that it is feasible to carry out the whole early warning of peanut aflatoxin. However, different regions of our country, different climates, impact factors and key control points are different. It is a long-term and efficient method to control aflatoxin pollution by systematically studying the critical control points of aflatoxin pollution in different producing areas and different links, establishing an early warning model, and early detection and early prevention.

## 5. Conclusions and Prospects

The strong toxicity and carcinogenicity of aflatoxin is a serious threat to human health and food safety. It is important to find a green, environmentally friendly and efficient means to effectively prevent and control aflatoxin contamination. In the long term, to ensure the quality and safety of agricultural products, it is also necessary to establish a full range of aflatoxin contamination early warning technology to control all aspects of the crop, to achieve the controllability of aflatoxin contamination, to achieve the full range of prevention and control from farm to fork, and to prevent and control mycotoxin contamination of agricultural product quality and safety. However, the model for China is established late, which needed to be based on different countries, different regions, different links, and different crops. The key control points are different, and the operability is more difficult. Although there are many ways for aflatoxin controlling, the biological control method has great advantages in terms of nutrients not being destroyed and not causing a lot of pollution. For example, the method for the introduction of biofungal agents in the soil not only effectively decreases the abundance of toxic aflatoxin-producing fungi, but also maintains the original quality of agricultural products. Moreover, it has more advantages of high safety, high efficiency and long persistence.

At present, a lot of research has been done on aflatoxin contamination control measures. Among them, the study of the interaction pattern between soil, plant and inter-root microorganisms provides new hints for the innovation of biological control methods of aflatoxin contamination in soil. The new direction of biological control measures for *A. flavus* in soil includes studying the distribution of microflora in different soil environments and resolving the interactions among inter-root microorganisms. Through risk assessment and early warning of contamination risk for crops in different growing regions, more data will be obtained to support the precise biological control of aflatoxin contamination in crops, thus improving the applicability of aflatoxin control mechanisms and reducing the losses caused by aflatoxin contamination.

## Figures and Tables

**Table 1:** Acceptable limits of aflatoxin in crops in several countries.

Countries	Crops	Limits (µg/kg)
China	cereals	20
USA	maize	5
EU	cereals	4
Japan	cereals	0
India	peanuts, maize	5

## Data Availability

Data available on request from the authors.

## Supplementary Materials

The following supporting information can be downloaded at: https://www.mdpi.com/article/10.3390/toxins15080475/s1, Table S1: types of post-harvest aflatoxin detoxification. References [81,82,83,84,85,86,87,88] are cited in Supplementary Materials.
