# Supplementary material for: Research Progress Related to Aflatoxin Contamination and Prevention and Control of Soils
Source: Toxins (Basel). 2023 Jul 25;15(8):475. doi: 10.3390/toxins15080475 (PMC10467090; doi:10.3390/toxins15080475)
Supplement: Supplementary file 1 [file toxins-15-00475-s001.zip › toxins-2419052-supplementary.pdf]

toxins

MDPI

Supplementary Materials: Research Progress Related to Aflatoxin Contamination and Prevention and Control of Soils

Xue Wang, Dun Wang, Shujuan Zhang, Mengjie Zhu, Qing Yang, Jing Dong, Qi Zhang and Peng Feng

Table S1. Types of post-harvest aflatoxin detoxification

Methods		Degradation rate	Application	Insufficient	Refs.
Physical methods	Mechanical methods	90%	Feed, food, etc.	Time consuming, resulting in a lot of waste	[81]
	Adsorption methods	92%	Feed, food, edible oil, etc.	Can attract nutrients and reduce value	[82]
	Glucomannan adsorption	100%	A compound detoxification agent is prepared and applied to feed to achieve in vitro detoxification of animals.	Not found	[83]
Chemical methods	Ozone treatment	93%	Used in small quantities in foods such as peanuts, cornmeal and chillies.	The treatment of small quantities is effective, but large quantities tend to change the nature of the food and deteriorate the taste, and the process is costly and time-consuming	[84]
	Ammonia treatment	95%	Used in the storage of grain in silos, this structure facilitates the use of ammonia fumigation and facilitates the recovery and removal of aflatoxin from food ingredients.	Ammonia can be harmful to human health and the environment, and can also damage the quality of food	[85]
	Essential oil fumigation	98%	Used on maize, rice, peanuts, etc. Simple and easy to use, suitable for home use and has no effect on the quality and nutritional content of the food.	Longer processing times, higher costs and non-recyclable	[86]
Biological methods	Bioenzyme digestion	96%	Used on peanut meal feed.	Additional additives are required to improve the detoxification effect and the treatment time is longer	[87]
	Microbial adsorption	99%	Suitable for use in feeds where certain nutrients can be added. Anaerobic culture of peanut meal with live lactic acid bacteria.	Harsh conditions	[88]
